# Wolf in Sheep’s Clothing: A Case of Sarcoidosis Exacerbating Asthma

**DOI:** 10.7759/cureus.110525

**Published:** 2026-06-09

**Authors:** Madeline Pan, Jennifer McCracken

**Affiliations:** 1 John Sealy School of Medicine, University of Texas Medical Branch at Galveston, Galveston, USA; 2 Allergy and Immunology, University of Texas Medical Branch at Galveston, Galveston, USA

**Keywords:** asthma, ground-glass opacities, non-necrotizing granulomas, pulmonary rehabilitation, sarcoidosis

## Abstract

As a leading cause of chronic respiratory morbidity worldwide, asthma remains a complex disorder characterized by inflammation, recurrent exacerbations, and episodic symptoms. Pulmonary sarcoidosis is a potential misdiagnosis for asthma. Symptoms such as shortness of breath, chest tightness, and dry cough can be present in both conditions, making differentiation difficult. This case highlights the diagnostic challenges and clinical overlap between severe asthma and sarcoidosis in a patient with uncontrolled respiratory symptoms, underscoring the importance of considering other etiologies when standard asthma management is ineffective in controlling symptoms. We present the case of a 59-year-old man with a history of severe asthma who continued to have persistent and worsening respiratory symptoms despite high-dose inhaled steroids, long-acting beta agonists (LABAs), long-acting antimuscarinics, and biologic therapy. Pulmonary function testing evolved from reversible obstruction to a more restrictive pattern, prompting imaging of his chest and subsequent lung biopsy, revealing noncaseating granulomas. This case highlights the diagnostic challenges and clinical overlap between severe asthma and sarcoidosis in a patient with uncontrolled and exacerbating respiratory symptoms.

## Introduction

Asthma is a heterogeneous disease characterized by chronic airway inflammation that affects over 300 million people across the world [1]. It is defined by the history of respiratory symptoms such as wheeze, shortness of breath, chest tightness, and cough [2]. Risk factors include a history of atopy and allergen exposure, genetics, early childhood viral infections, tobacco smoke exposure, vitamin D deficiency, etc. [3]. Initial therapy is inhaled corticosteroids (ICS), with step-up treatment to include long-acting beta agonists (LABAs) and long-acting muscarinic agonists (LAMAs) depending on severity [2]. 

Sarcoidosis is a systemic granulomatous inflammatory disease that affects the lungs and/or lymph nodes in over 90% of affected individuals [4]. There is no gold standard test for the diagnosis of sarcoidosis; therefore, high clinical suspicion is required, and other mimickers must be excluded, including infections such as mycobacteria and fungi, malignancy, and environmental/occupational exposures causing pulmonary manifestations [5]. Systemic corticosteroids are first-line therapy for patients with sarcoidosis. 

Because cough, wheezing, and chest pain may occur in both conditions, diagnostic evaluation with chest imaging, pulmonary function testing, and empiric therapeutic trials may aid in distinguishing between them. A comprehensive clinical history, including assessment of atopy, allergen exposures, pulmonary symptoms, and family history of pulmonary disease, may also provide valuable information to help differentiate these entities. 

## Case presentation

A 59-year-old man with a past medical history significant for herpes zoster (2017), hypertension, third-degree heart block status post permanent pacemaker placement, and chronic rhinitis presented to the allergy and immunology clinic for evaluation and management of severe asthma. He reported a longstanding history of asthma since childhood that had remained poorly controlled. At presentation, he endorsed daily dyspnea associated with productive cough, chest tightness, and wheezing. His dyspnea was exacerbated by exertion and when lying supine. 

Before presentation, the patient had relied primarily on over-the-counter epinephrine inhalation aerosol (Primatene Mist) several times weekly for symptom relief. More recently, he had been initiated on budesonide-formoterol 160/4.5 mcg, two puffs daily, with partial symptomatic improvement. He denied any personal smoking history but reported substantial secondhand smoke exposure as well as prior occupational asbestos exposure while working as an engineer. Symptoms did not worsen during the work week. 

Initial spirometry (Table 1) demonstrated a severe obstructive ventilatory defect with reduced forced vital capacity (FVC) and forced expiratory volume in 1 second (FEV1), with significant bronchodilator response. These findings, in conjunction with his clinical presentation, were concerning for uncontrolled severe persistent asthma. At the initial clinic visit, his budesonide-formoterol dose was increased to two puffs twice daily. 

**Table 1:** PFT results This table shows a low FEV1, low FVC, and reduced ratio with reversibility indicative of obstruction and consistent with asthma. However, one year later, a repeat PFT showed low FEV1, low FVC, but a reduced ratio without reversibility. FEV1: forced expiratory volume; FVC: forced vital capacity; TLC: total lung capacity; PFT: pulmonary function test

Test Occasion	Initial PFT	Follow-Up PFT
FEV1 in Liters	1.52 (39%)	1.42 (39%)
FVC in Liters	2.74 (55%)	2.37 (51%)
FEV1/FVC (%)	56	60
FEV1 Percent Change Following Bronchodilator Use (%)	16	4
TLC		4.41 (59%)

Follow-up was delayed due to financial issues, and he returned to the clinic a year later, reporting his symptoms had significantly worsened despite remaining adherent to his prescribed budesonide-formoterol dose. Tiotropium was added to his medications, and phenotyping for his asthma was performed. Repeat spirometry (Table 1) revealed a combined obstructive and restrictive ventilatory defect with a non-significant bronchodilator response (FEV1 improvement less than 12% and less than 200 mL). Fractional exhaled nitric oxide (FeNO) measured 15 ppb, which was within normal limits (<25 ppb). Alpha-1 antitrypsin levels were also within the normal reference range. Total IgE was 24 kU/L, and CBC showed an eosinophil count of 120 cells/μL. Treatment with high-dose ICS-LABA and tiotropium provided minimal benefit. In accordance with the Global Initiative for Asthma Guidelines, he stepped up therapy and started treatment with the biologic tezepelumab 210 mg every four weeks, but symptoms of dyspnea on exertion worsened [2]. Computed tomography (CT) of the chest (Figure 1) was ordered and demonstrated diffuse bilateral ground-glass opacities, raising concern for an additional underlying etiology contributing to the patient’s respiratory symptoms. He was referred to the pulmonologist for further evaluation with a bronchoscopy and bronchoalveolar lavage. Respiratory cultures and special stains for acid-fast bacilli and fungi were negative. Cytology revealed no malignant cells, and PCR for *Mycobacterium tuberculosis *was negative. Body fluid cell count showed 16% lymphocytes.

**Figure 1:**
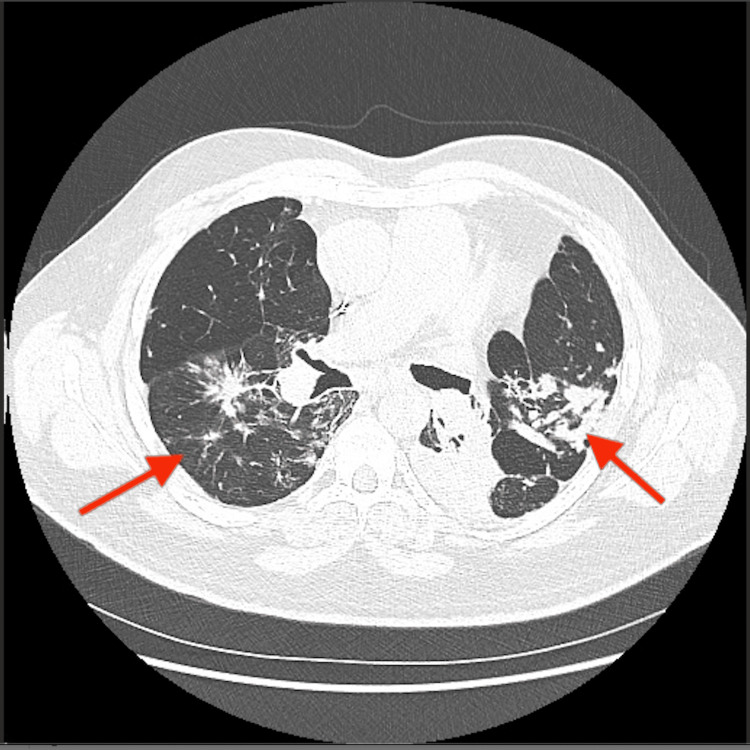
Computed tomography (CT) scan illustrating numerous small foci of nodularity with ground glass opacity along with centrally calcified mediastinal and hilar lymph nodes The arrows point to foci of nodularity with ground-glass opacities present in the lungs bilaterally. Common CT findings in sarcoidosis include bilateral symmetric hilar and mediastinal lymphadenopathy, which may demonstrate calcification, as well as pulmonary micronodules and peribronchovascular thickening. In more advanced stages of disease, fibrotic changes may also be observed.

An open lung biopsy was subsequently performed following nondiagnostic transbronchial biopsy findings. Histopathologic examination demonstrated multiple well-formed non-necrotizing granulomas with surrounding dense hyalinizing fibrosis (Figure 2 and Figure 3). The morphological and architectural appearance of the lesions was consistent with fibrosing sarcoidosis. He was diagnosed with sarcoidosis and started on oral prednisone 20 mg daily while continuing the ICS-LABA and tiotropium. Tezepelumab was discontinued due to a lack of benefit and an alternative diagnosis. The patient was referred for ophthalmologic and cardiac evaluation to rule out evidence of ocular and cardiac sarcoidosis, but he did not attend. Prednisone 20 mg daily was continued for two months, then slowly tapered to 15 mg daily for one month, 10 mg daily for one month, 7.5 mg daily for one month, 5 mg daily for one month, and 2.5 mg daily for one month. Symptoms have remained stable while continuing pulmonary rehabilitation and taking budesonide-formoterol 160-4.5 mcg (two puffs twice a day) and tiotropium 2.5 mcg daily. 

**Figure 2:**
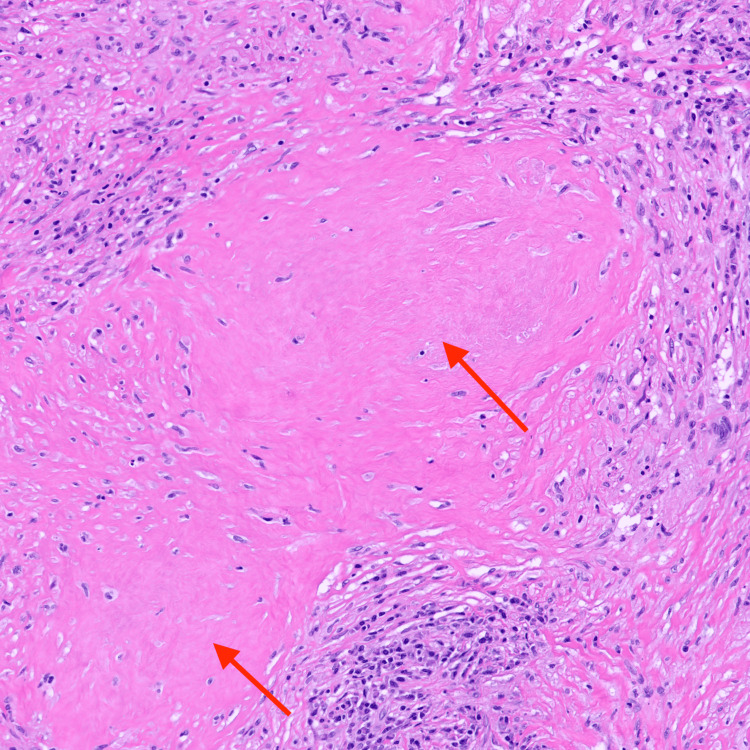
Central bronchus biopsy Histology indicative of dense fibrotic areas with lymphoplasmacytic and histiocytic inflammation. Red arrows indicate areas of dense collagen (fibrosis) surrounded by prominent lymphoplasmacytic and histocytic infiltrate. This infiltrate typically surrounds granulomas, which are associated with fibrosis. This combination is supportive of sarcoidosis.

**Figure 3:**
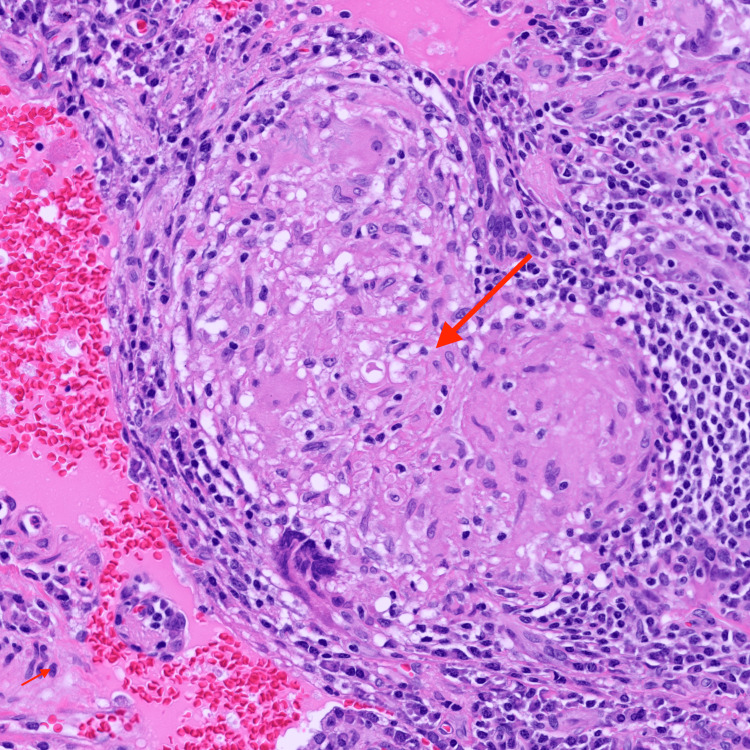
Central bronchus biopsy Pathology indicative of non-necrotizing granulomas present in the lungs, which is a suggestive feature of inflammatory diseases, such as sarcoidosis. The arrow points to a noncaseating granuloma.

In the most recent outpatient follow-up, the patient reported stable respiratory symptoms without significant worsening. He reported good adherence to his medication regimen of ICS-LABA and tiotropium. Recent PFT indicated some decline in FEV1, but otherwise stable.  

## Discussion

This case underscores the substantial clinical overlap between asthma and sarcoidosis, which may complicate timely and accurate diagnosis. The patient presented with dyspnea, cough, and chest tightness, symptoms that are common to both conditions. In patients with presumed asthma who exhibit atypical features or an incomplete response to standard therapy, the possibility of an alternative or coexisting pulmonary disorder should be carefully considered. 

Initially, the patient’s history of poorly controlled childhood asthma, spirometry demonstrating an obstructive ventilatory defect with significant bronchodilator responsiveness, and partial symptomatic improvement with ICS-LABA therapy supported a working diagnosis of uncontrolled asthma [6]. However, subsequent clinical evolution prompted reassessment. Repeat pulmonary function testing revealed a mixed obstructive and restrictive pattern without bronchodilator reversibility, findings that are not characteristic of asthma alone. In addition, asthma-related biomarkers, including total IgE, peripheral eosinophil count, and FeNO, were unremarkable, arguing against a type 2-high inflammatory phenotype [7]. The absence of sustained clinical improvement despite escalation of inhaled therapy and initiation of biologic treatment with tezepelumab further heightened concern for an alternative or concurrent pulmonary process. 

CT imaging demonstrated diffuse ground-glass opacities and centrally calcified mediastinal and hilar lymphadenopathy, findings suggestive of prior granulomatous disease with possible active inflammatory or interstitial lung involvement [8]. Comprehensive infectious evaluation, including testing for *Mycobacterium tuberculosis*, histoplasmosis, and other fungal pathogens, was negative. Ultimately, tissue diagnosis was required for diagnostic clarification. Transbronchial biopsy was nondiagnostic, and subsequent open lung biopsy demonstrated non-necrotizing granulomas. The differential diagnosis for non-necrotizing granulomatous inflammation is broad and includes sarcoidosis, hypersensitivity pneumonitis, berylliosis, granulomatosis with polyangiitis, foreign body reactions, fungal infections, mycobacterial disease, and malignancy [9]. The absence of identifiable infectious organisms and negative microbiologic studies substantially reduced the likelihood of infectious etiologies. Additionally, the relative stability of radiographic findings over time made metastatic disease less likely. 

This case highlights the recognized clinical challenge posed by overlapping or ambiguous presentations of asthma and sarcoidosis. Currently, there is no single test that reliably confirms or excludes asthma from sarcoidosis. Therefore, the clinical and laboratory results play a role in differentiating between the two conditions. Exacerbations associated with worsening extrapulmonary disease, exacerbations associated with new/worsening infiltrates on lung imaging, restrictive ventilatory defect, and elevated serum angiotensin-converting enzyme (ACE) level are distinguishing features of sarcoidosis. Meanwhile, a patient having a history of atopy, family history of asthma, and exacerbations associated with common asthma triggers points more towards asthma. Additionally, detecting an elevated eNO, elevated serum IgE level, peripheral eosinophilia, and induced sputum eosinophilia also align more with asthma [10]. Presence of a positive bronchial challenge test is not a definitive result, as it is suspected that bronchial hyperresponsiveness is prevalent in asthma and sarcoidosis [11]. 

These diagnostic challenges can lead to delays in treatment or overdiagnoses of asthma in sarcoidosis patients. Corticosteroids are the first-line treatment of sarcoidosis, with the initial dose tapered after three to six weeks to a target maintenance dose of 5-10 mg prednisone by six months of treatment. [12] In contrast, management of asthma is dependent on frequency and severity of symptoms, starting first with a low dose of ICS-formoterol as needed. If symptoms are not well-controlled, the dose of the ICS-LABA can be increased [2]. Further escalation therapies include LAMAs and biologic agents such as anti-IgE, anti-IL5/5R, anti-IL4/IL-13, or anti-TSLP, like tezepelumab, in patients with refractory symptoms. It is important to note that oral corticosteroids can be used to treat both sarcoidosis and asthma exacerbations. Therefore, response to treatment alone is insufficient to distinguish between the two conditions, particularly because patients with sarcoidosis-associated bronchial hyperresponsiveness may also benefit from ICS-LABA therapy. 

When asthma cannot be clearly established or excluded in a patient with sarcoidosis, Kalkanis and Judson suggest initiating empiric therapy with systemic corticosteroids (prednisone 20 mg daily) and reassessing symptoms after two to three weeks [10]. If the patient experiences significant improvement, corticosteroids can be gradually tapered; recurrence of pulmonary symptoms below a certain steroid dose threshold favors sarcoidosis as the underlying condition. In contrast, a lack of improvement may suggest poorly controlled asthma (which often requires higher doses of oral steroids to treat exacerbations), in which case combined ICS-LABA therapy may provide benefit. In this patient, the clinical response to systemic corticosteroids and histopathologic confirmation ultimately supported sarcoidosis as the primary etiology. 

## Conclusions

In conclusion, this case underscores the important diagnostic challenge posed by overlapping clinical features of asthma and pulmonary sarcoidosis, particularly in patients with persistent symptoms despite guideline-directed therapy. Our patient’s clinical course highlights that a lack of sustained response to escalating asthma treatment should prompt reconsideration of the diagnosis and evaluation for alternative or coexisting pulmonary conditions. Sarcoidosis frequently mimics asthma with shared symptoms such as dyspnea, cough, and wheezing, while bronchial hyperresponsiveness and obstructive physiology may further confound diagnosis and lead to overdiagnosis of asthma or delayed recognition of sarcoidosis. Importantly, no single diagnostic test reliably distinguishes these conditions, and even commonly used tools such as bronchial provocation testing may be inconclusive in this context. This reinforces the necessity of a comprehensive, longitudinal approach incorporating pulmonary function trends, imaging, and, when needed, histopathologic confirmation. Early recognition of alternative etiologies is essential to avoid unnecessary treatment, reduce healthcare burden, and ensure initiation of appropriate therapy, such as systemic corticosteroids for sarcoidosis. Ultimately, this case highlights the need for heightened clinical vigilance and continued research to better define diagnostic strategies for patients with overlapping obstructive and granulomatous lung disease. 
